# Current Difficulties for General Practitioners in the Diagnosis and Management of Long COVID Patients: A Cross-Sectional Study Assessing an Online Questionnaire

**DOI:** 10.3390/jcm15082855

**Published:** 2026-04-09

**Authors:** Cléa Le Breton, Timothée Klopfenstein, Souheil Zayet

**Affiliations:** 1 Infectious Diseases and Tropical Medicine Department, Nord-Franche-Comté Hospital, 90400 Trevenans, France; timothee.klopfenstein@hnfc.fr; 2 Clinical Research Unit, Nord-Franche-Comté Hospital, 90400 Trevenans, France; 3 Infectious Diseases, Infection Prevention and Control Department, Hôpitaux Robert Schuman, 2540 Luxembourg City, Luxembourg

**Keywords:** long COVID, post-acute sequelae of COVID-19, general practitioners, difficulties, Franche-Comté

## Abstract

**Background:** Long COVID presents a novel and emerging public health challenge. As the first point of contact, general practitioners (GPs) play a key role in diagnosing and coordinating the care of patients presenting with post-acute sequelae of COVID-19 (PASC), despite a lack of experience. This study aimed to identify the main difficulties encountered by GPs in Franche-Comté, France, in managing adult outpatients with long COVID. **Methods:** We conducted a cross-sectional survey using an anonymous online questionnaire, which contained 21 questions and was distributed to GPs in Franche-Comté, France. The survey assessed definition, diagnostic and therapeutic challenges in managing long COVID. **Results:** Among the 410 questionnaires distributed, 90 general practitioners (GPs) responded (response rate: 21.9%). The mean age of participants was 34 ± 10 years, and 64.4% were women (*n* = 58). Regarding knowledge of long COVID, three participants (3.3%) did not recognize it as a distinct clinical entity, while more than half (58.9%, *n* = 53) reported insufficient knowledge. The main challenges identified were therapeutic management (76.7%, *n* = 69) and diagnosis (75.6%, *n* = 68). Only 4.5% of respondents (*n* = 4) reported no difficulty in defining post-acute sequelae of SARS-CoV-2 infection (PASC). The most frequently reported diagnostic difficulty was distinguishing long COVID from differential diagnoses (93.3%, *n* = 83/89), particularly fibromyalgia (94.3%, *n* = 83/88). Only 37.1% of participants (*n* = 33/89) reported actively following up patients with PASC. During initial management, the main challenge was the difficulty in objectively assessing patients’ complaints using available diagnostic tools (80.7%, *n* = 67/83). Additionally, a large majority of GPs reported difficulties in addressing patients’ questions (86.7%, *n* = 72/83) and managing associated anxiety disorders (75.9%, *n* = 63/83). **Conclusions:** These findings highlight the immediate need to enhance GP training in Franche-Comté, France, in dealing with long COVID. Improvements such as harmonizing long COVID definitions, testing diagnoses, and strengthening interdisciplinary coordination are essential to provide coherent and patient-centered care for this disease.

## 1. Introduction

Since its first appearance in China in December 2019, severe acute respiratory syndrome coronavirus 2 (SARS-CoV-2) [1,2] spread worldwide, causing a large global outbreak and pandemic declared by the World Health Organization (WHO) in March 2020 [3].

Coronavirus Disease 2019 (COVID-19), caused by the SARS-CoV-2 virus, can cause mild to severe respiratory illness. The acute COVID-19 phase is currently well defined. It typically lasts up to 4 weeks from the onset of initial infection. However, emerging evidence indicates that SARS-CoV-2 has also short- and potential long-term adverse health outcomes. Several COVID-19 patients have reported persistent symptoms, lasting for weeks or months after the acute phase, with recurrences, that sometimes impact quality of life [4,5,6].

In October 2021, the WHO defined the post-COVID-19 condition as the continuation or development of new symptoms 3 months after the initial SARS-CoV-2 infection, with these symptoms lasting for at least 2 months, with no other explanation. Symptoms may be new-onset following initial recovery from an acute COVID-19 episode or persist from the initial illness; symptoms may also fluctuate or relapse over time [7].

Long COVID or post-acute sequelae of COVID-19 (PASC) [8] is not the first reported post-acute infection syndrome (PAIS). Other viral (Ebola [8,9], polioviruses [10], arboviruses such as dengue [11,12] and chikungunya [13], Epstein–Barr virus (EBV) and other coronaviruses [14]) and bacterial infections (*Streptococcus pyogenes*, *Campylobacter jejuni* [15], *Mycoplasma pneumoniae*, and *Coxiella burnetii* [16]) are also linked to long-term consequences [8,16,17] with a substantial healthcare burden. These PAISs, including long COVID, show highly heterogeneous symptoms [8,17,18], and their underlying mechanisms remain poorly understood.

One of the major limitations in long COVID management is the recognition of the disease. Long COVID syndrome recognition and management are priorities requiring effective management in primary care in France. Patients have often been under-diagnosed and given a diagnosis confused with several differential diagnoses such as fibromyalgia, irritable bowel syndrome or Lyme disease [9]. Currently, more than 200 symptoms have been identified, with impacts on multiple organ systems, such as fatigue, brain fog, and cardiovascular or neurological problems, as well as psychological distress and no specific clinical features [8,10,11]. There are also no useful or specific laboratory and/or imaging tests to diagnose long COVID. For each patient identified with long COVID, the approach should be multidisciplinary and is most often coordinated by the patient’s general practitioner (GP). This approach should also require collaboration with other healthcare professionals, with patient-centered care [12,13].

France has a universal healthcare system primarily funded through public health insurance, ensuring broad access to medical services for the population. A mix of public and private providers characterizes the system, with patients benefiting from a high degree of choice and relatively low out-of-pocket costs. GPs play a central role as the first point of contact within the healthcare system and act as gatekeepers to specialized care. They are responsible for coordinating patient care, managing chronic conditions, and providing preventive services, thereby contributing to the overall efficiency and continuity of care. Indeed, the role of GPs is crucial in the management of PAISs, including long COVID [14], with supporting care-coordinator expertise [15,16,17,18,19]. In March 2022, Santé publique France estimated the prevalence of long COVID to be 4% in the general French adult population [20]. In our region (Franche-Comté) and hospital, we have observed a delay in the diagnosis of long COVID patients referred by GPs, probably due to a lack of information.

Therefore, the aim of our study was to examine GP experiences of caring for outpatients with long COVID in Franche-Comté, France, to explore difficulties in PASC diagnosis and therapeutic management and finally to identify unmet care needs of GPs.

## 2. Material and Methods

### 2.1. Study Design

We conducted a cross-sectional descriptive study concerning the difficulties in the diagnosis and therapeutic management of adult outpatients with long COVID among GPs in Franche-Comté, France. An online anonymous questionnaire was formulated following the scientific literature on long COVID. The questionnaire was validated in January 2025 by a panel of experts consisting of two Infectious Diseases specialists from Nord Franche-Comté Hospital and two Professors in General Medicine from Besançon Medical University, France. The questionnaire contained 21 questions with 16 multiple-choice and 5 open-ended questions. There was no pilot test of the survey before its distribution.

An email containing information about this study and providing an URL (Supplementary Material File S1) to the electronic survey was sent to practicing GPs in Franche-Comté, France. The survey was launched though (i) a mailing list from the Department of General Medicine at the Besancon Faculty of Medicine, France; (ii) regional professional health communities in Franche-Comté, France; and (iii) direct contact with GPs. Two reminders were sent in the case of no response.

### 2.2. Data Collection and Study Period

The questionnaire had four sections: (i) baseline characteristics and epidemiologic data of participants and (ii) GP difficulties with long COVID definition, (iii) diagnosis and (iiii) therapeutic management. An additional section allowed participants to make suggestions for the improvement of care for patients with PASC in Franche-Comté, France. GPs were invited to participate in this study on a voluntary basis. To characterize GPs, demographic characteristics (sex and age) and county-specific practice characteristics (practice region and practice type) were collected. No identifiable or personal data was collected. First, we asked GPs in general/global terms what difficulties they encountered with long COVID, before addressing more specific aspects in the rest of the questionnaire. After answering all the questions, the GP has access to a summary sheet covering the key points of long COVID in adults (Supplementary Material File S2). All survey data were collected using the online questionnaire; the study inclusion period was 6 weeks (1 February to 15 March 2025).

### 2.3. Definitions (Supplementary Material File S3)

Definitions were not included in the survey.

### 2.4. Data Analysis

We expressed discrete variables as numbers and percentages, and continuous variables as means and standard deviations (SDs).

## 3. Results

### 3.1. Baseline Characteristics and Epidemiologic Data

**Figure 1:**
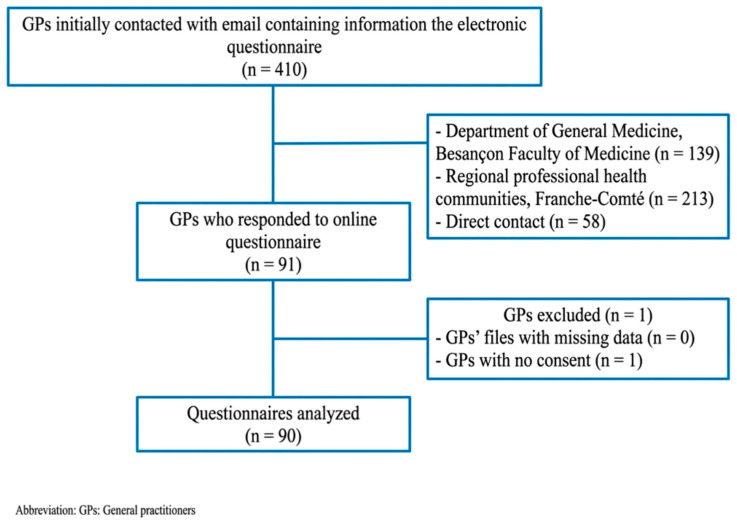
Flow chart.

In this study, 410 GPs were initially contacted with an email containing information about the electronic questionnaire (among a total of 3400 GPs in the region approximately). Then, 90 questionnaires (21.9%) were completed, received and then analyzed (Figure 1).

Among the included GPs, we noted a female predominance (*n* = 58, 64.4%) (Table 1 and Table 2). The mean age of the participants (SD) was 34 (±10.5), with more than three-quarters under 40 years (Table 1). GPs over 40 years were more often female and predominantly established in regular practice, whereas those under 40 were more frequently in training or working as substitutes.

More than half of the participants were young practitioners, with 25 (27.7%) students in their final year of general practice and 23 (25.6%) locums. There was an over-representation of GPs in the Doubs department (*n* = 46, 51.1%) (Supplementary Material File S4).

### 3.2. Definition and Global Difficulties

**Figure 2:**
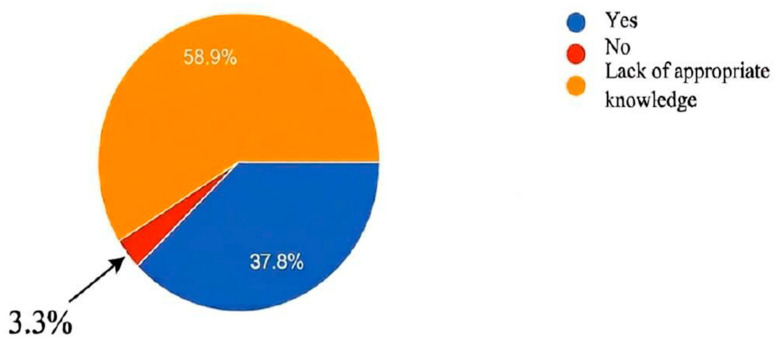
Repartition of disease recognition of long COVID in participants.

Fifty-three of the GPs responding (58.9%) confirmed that they had insufficient knowledge of long COVID. However, 3.3% of participating GPs (*n* = 3) did not recognize this disease as a real entity (Figure 2).

The two main challenges for GPs (*n* = 90) were long COVID therapeutic management and diagnosis (*n* = 69, 76.7%, and *n* = 68, 75.6%), respectively. Additionally, more than half of the participants also highlighted difficulties with social support (*n* = 52, 57.8%) and with the long COVID global definition (*n* = 50, 55.6%). Eighty-five participants from 89 GP centers (95.5%) reported at least one challenge concerning difficulties in accurately defining “long COVID”, which are detailed in Table 3.

### 3.3. Diagnosis Difficulties

All participants admitted to having at least one difficulty or more with confirming or ruling out the diagnosis (Table 4).

The main limiting factor was clinical polymorphism, with several differential diagnoses (*n* = 83/89, 93.3%), such as fibromyalgia (*n* = 83/88, 94.3%), which was the main one. We also noted that more than three-quarters of GPs (*n* = 69/89, 77.5%) surveyed reported diagnostic difficulties when managing patients with a medical past history of depression, anxiety and/or post-traumatic stress disorders.

However, the difficulty of obtaining a specialist’s opinion and access to additional tests were mentioned less frequently (*n* = 34/89, 38.2%, and *n* = 20/89, 22.5%, respectively).

### 3.4. Therapeutic Management Difficulties

Only 33 participants out of 89 responding to this section (37.1%), which reported follow-up with patients with long COVID (Table 5).

During initial management, the most frequently reported difficulty, cited by 67/83 GPs (80.7%), was identifying patients’ complaints. Additionally, 52/83 (62.7%) of them mentioned that the lack of documents or information support on long COVID patient care was also a limitation. Approximately half of them also deplored the lack of recognition by other specialists, and the difficulty in referring patients to specialized centers (*n* = 41/83, 49.4% for each one), which are not available in Franche-Comté, France.

Regarding treatment initiation, the main constraint for 67/82 (81.7%) participants was the lack of a well-defined consensus to which GPs can relate. About two-thirds of GPs (*n* = 53/83, 64.6%) also highlighted clinical polymorphism as a limiting factor in personalized treatment initiation, leading to difficulties in prioritizing therapeutic approaches for 22 GPs (26.8%).

When dealing with patients, a large majority of GPs (*n* = 72/83, 86.7%) reported difficulties in answering patients’ questions, with 63/83 (75.9%) finding it challenging to manage their anxiety disorders. Only 20 (24.1%) reported difficulties in establishing a therapeutic alliance.

### 3.5. Participants’ Suggestions

**Figure 3:**
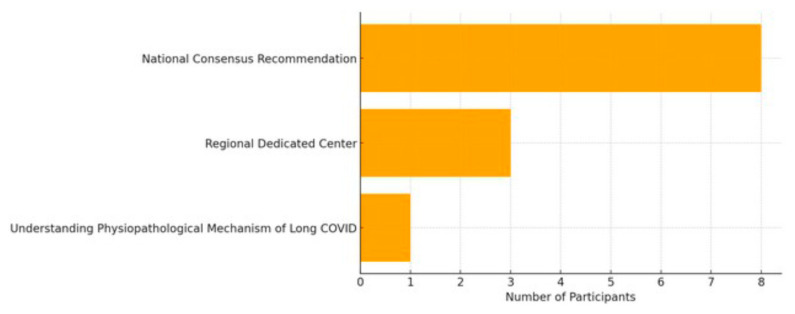
Different general practitioners’ suggestions.

Furthermore, 12 GPs (13.3%) provided suggestions for improvements for supporting long COVID patients in their work in Franche-Comté, France (Figure 3).

## 4. Discussion (Supplementary Material File S5)

### 4.1. Baseline Characteristics and Epidemiologic Data

The response rate of 21.9% (90 out of 410 GPs) is relatively low for GP participants. This can be explained by the high workload and time pressure, the format issue of the online questionnaire and the fact that many GPs receive frequent survey requests, which can make them less motivated to respond. This low result may not represent the whole GP population in our department. This leads to several possible non-response biases such as selection bias, over-representation of certain views and under-representation of busy or older practitioners.

There was delayed recognition by the medical establishment of PASC as a clinically significant entity, with a small or minor proportion of GPs still denying this disease altogether [21,22]. In Franche-Comté, France, the majority of GPs who responded to our survey acknowledged long COVID as a legitimate or real condition. Illness denial was reported only by three GPs. Despite this, more than half reported a significant lack of knowledge and a poor understanding of long COVID.

Furthermore, we observed concerning figures regarding their difficulties in both diagnosing and managing the disease as a whole.

The relatively young mean age of our participants suggests a more recent medical degree, probably with less experience in outpatient diagnosis and therapeutic management. It is also possible that younger GPs were more inclined to respond to the questionnaire because they are currently writing or have recently completed a thesis, making them more open to scientific research. This must also be interpreted in the context of the recently recognized condition of long COVID. Participating physicians might have a specific interest in long COVID, leading to skewed findings. Only 37.1% of respondents reported treating long COVID patients, which raises concerns in our department and is a real limitation of this study. This can also be explained by the predominance of young GPs who have insufficient professional experience to deal with this complex disease.

The predominance of female respondents in our study may reflect the growing feminization of the medical profession, already well documented in health education and training programs in France [23].

There is also an over-representation of GPs working in the Doubs department (51.1%). This may be explained by the presence of the regional University hospital in Besançon in this department; professionals are more connected to the University and more frequently invited to participate in studies. Additionally, the higher number of GPs in this department compared to the others could also be a contributing factor.

### 4.2. Definition and Global Difficulties

Our participants expressed concern over the absence of a single, consensual definition of long COVID. Confusion persists regarding the onset and duration of symptoms, as well as diagnostic criteria. This issue has also been highlighted in other studies. In an Austrian study exploring GPs’ perceptions and experiences of long COVID, participants criticized the discrepancies between the WHO and National institute for Health and Care Excellence definitions and the challenges of applying them in general practice [22], which is the case in our study regarding the divergence between the consensual definitions (WHO and French National Health Authority). Similarly, Irish studies from 2022 [15] and 2023 [17] reported that GPs emphasized the need for a more standardized approach and specific training, despite the fact that the WHO definition had already been established.

Coordination with other healthcare professionals was reported as a difficulty by less than half of our participants (43.3%), a point that will be further elaborated later in the Discussion Section.

### 4.3. Diagnosis Difficulties

More than three-quarters of GPs reported difficulties in establishing a diagnosis when asked at first about the challenges encountered in long COVID. All participants acknowledged at least one limitation in accurately diagnosing the disease when asked more specifically about diagnosis barriers.

The primary challenge identified in our study was the clinical polymorphism of long COVID, which involves a broad range of symptoms and several differential diagnoses. This complexity is further complicated in patients with pre-existing or associated mental health disorders, as noted by several practitioners. In the Netherlands, GPs concluded that comorbidities significantly increasing PASC risk included mental illness [24], with a possible long-term impact.

Additionally, 70% of participants highlighted insufficient training in the use of available diagnostic testing, including both laboratory and imaging tests.

Although healthcare system differences could affect GPs’ experiences, our data were consistent with other European studies conducted in different countries. GPs reported a lack of reliable information on diagnostic procedures in a study conducted in Malta and Belgium in 2022 [19]. Similarly, a study in Ireland in 2023 concluded that 92.45% of participants lacked confidence in diagnosing long COVID, and most were unsure how to manage and organize its diagnosis [21] and treatment [17].

Few participants mentioned limited access to diagnostic tests (22.5%) or specialist consultations (38.2%) as a difficulty. However, in the section concerning treatment-related challenges, limited access to other healthcare professionals (45.1%) and long waiting times for specialized care (65.9%) were more frequently reported. The study highlights a lack of teamwork among healthcare providers as a problem. Only 43.3% of GPs reported issues with colleagues, which may indicate a lack of awareness about the need for better coordination. In a region such as ours, known for its high prevalence of Lyme neuroborreliosis [20], this is also evident in the coordination between general practitioners and hospitals to establish a structured care plan.

As known, GPs are often present on the front line for suspecting and diagnosing complex conditions such as long COVID. Enhanced collaboration between primary and secondary care could improve access to specialized diagnostic investigations.

For example, in PASC patients with neurological presentation, lumbar puncture (performed at the hospital) is essential for diagnosing Lyme neuroborreliosis [20] or infectious encephalitis [25].

### 4.4. Treatment Difficulties

As previously reported, there was discordance in the collected results concerning some questions that were raised. When GPs were initially asked about their overall difficulties in managing patients with long COVID, 76.7% participants reported treatment-related challenges. However, when the question was later reformulated to focus more specifically on the initial phase of care, 92.2% GPs acknowledged difficulties.

The main challenge identified by GPs was the difficulty in identifying patients’ symptoms through diagnostic testing, often compounded by limited access to reliable informational resources. While clinical polymorphism was less frequently cited as a barrier during treatment than during diagnosis, it remained a concern for many. Despite this, only a minority of GPs reported difficulties in prioritizing therapeutic strategies.

Regarding the GP–patient relationship, most participants reported a satisfactory therapeutic alliance. Nevertheless, many experienced difficulties in addressing patients’ questions and managing their anxiety.

The central role of GPs as the first point of contact and as care coordinators was clearly defined in a qualitative German study conducted between 2022 and 2023, which focused on the management of PASC in general practice [18]. The study revealed a sense of helplessness among GPs, primarily due to the limited availability of testing for managing such conditions in primary care, as well as restricted access to secondary care services—particularly mental health support—often associated with long waiting times. These findings are consistent and similar to our study: 45.1% participants reported difficulties in accessing other healthcare professionals, and 65.9% cited prolonged waiting times as a major barrier.

### 4.5. Limitations

There are a few limitations in our study: (i) The size of our sample and the limited number of participants means it is not representative on a regional and national scale. Currently, there are 241,255 practicing physicians in France, 42.3% whom are GPs [26]. (ii) This design also introduces a selection bias, as physicians who chose to participate may have had a particular interest in the topic of long COVID and therefore may not accurately reflect the broader scope of general practice. The study’s cross-sectional design restricts understanding of how GPs’ knowledge and attitudes may have changed over time regarding long COVID. This design can also distort results by introducing confounding factors and unrepresentative samples, leading to misleading associations rather than true causal effects.

Qualitative research could complement the current findings by exploring GPs’ experiences and patient perspectives in more depth.

(iii) In our Discussion Section, our review of European countries reporting the same topic did not respect PRISMA guidelines. (iiii) Finally, many of the GPs included in our study had never managed patients with long COVID. In fact, only 33 respondents reported actively following up such patients, with a relatively high proportion of physicians who are still in training, which corresponds to the current state of general medicine in France.

## 5. Conclusions

This study aimed to highlight the difficulties reported by GPs in Franche-Comté, France, in managing PASC patients.

Long COVID remains insufficiently defined and poorly understood by many GPs. Its clinical variability, the absence of standardized guidelines, and limited access to specialist units contribute to major diagnostic and therapeutic challenges. Moreover, patient anxiety and the high request for information made the GP–patient relationship vulnerable.

Improving long COVID care will require targeted and immediate actions: developing practical, evidence-based training for GPs in Franche-Comté; aligning national definitions and creating diagnostic algorithms and regional long COVID clinics; and better access to mental health and rehabilitation services. In order to generalize these conclusions, we recognize the need for larger national studies or studies in a broader European context.

## Figures and Tables

**Table 1:** Baseline characteristics and epidemiologic data of participants.

Characteristics	All GPs (*n*)	Percentage (%)
Sex		
Male	32	35.6
Female	58	64.4
Age (y)		
<30	39	43.3
30–39	30	33.3
40–49	12	13.3
50–59	6	6.8
>60	3	3.3
Professional title		
Ambulatory training in PC with supervised autonomy	25	27.7
Substitute GPs	23	25.6
GPs	42	46.7
Practice setting		
Rural	28	31.1
Urban	17	18.9
Semi-rural	45	50
Department		
(25)—Doubs	46	51.1
(39)—Jura	11	12.2
(70)—Haute Saône	10	11.1
(90)—Territoire de Belfort	23	25.6

Abbreviations: GPs: General practitioners; *n*: number; PC: primary care; y: years.

**Table 2:** Comparative table of general practioners under and over 40 years of age.

Characteristic	Overall, N = 89 ^1^	GPs > 40 y (N = 21 ^1^)	GPs < 40 y (N = 68 ^1^) *	*p*-Value ^2^
Sex				0.018
Male	32.0 (36%)	3.0 (14%)	29.0 (43%)	
Female	57.0 (64%)	18.0 (86%)	39.0 (57%)	
**Age (y)**				<0.001
<30	39.0 (44%)	0.0 (0%)	39.0 (57%)	
>60	3.0 (3%)	3.0 (14%)	0.0 (0%)	
30–39	29.0 (33%)	0.0 (0%)	29.0 (43%)	
40–49	12.0 (13%)	12.0 (57%)	0.0 (0%)	
50–59	6.0 (7%)	6.0 (29%)	0.0 (0%)	
**Professional title**				<0.001
Ambulatory training in PC with supervised autonomy	25.0 (28%)	0.0 (0%)	25.0 (37%)	
GPs	41.0 (46%)	20.0 (95%)	21.0 (31%)	
Substitute GPs	23.0 (26%)	1.0 (5%)	22.0 (32%)	
**Practice setting**				0.10
Rural	28.0 (31%)	3.0 (14%)	25.0 (37%)	
Semi rural	45.0 (51%)	12.0 (57%)	33.0 (49%)	
Urbain	16.0 (18%)	6.0 (29%)	10.0 (15%)	
**Department**				0.7
25—Doubs	46.0 (52%)	9.0 (43%)	37.0 (54%)	
39—Jura	11.0 (12%)	2.0 (10%)	9.0 (13%)	
70—Haute-Saône	10.0 (11%)	3.0 (14%)	7.0 (10%)	
90—Territoire de Belfort	22.0 (25%)	7.0 (33%)	15.0 (22%)	
**Recognition of the disease**				0.5
Lack of sufficient knowledge	53.0 (60%)	11.0 (52%)	42.0 (62%)	
No	3.0 (3%)	0.0 (0%)	3.0 (4%)	
Yes	33.0 (37%)	10.0 (48%)	23.0 (34%)	
**Managing patients with Long COVID**				0.080
No	56.0 (64%)	10.0 (48%)	46.0 (69%)	
Yes	32.0 (36%)	11.0 (52%)	21.0 (31%)	

^1^ *n* (%); ^2^ Pearson’s Chi-squared test; Fisher’s exact test. Abbreviations: PC: primary care, y: years. * One GP was excluded from this comparison because the data was missing.

**Table 3:** Definition and global difficulties of participants in patients with long COVID.

Question	All GPs (*n*)	Percentage (%)
**On what issues are you experiencing/encountering considerable difficulties?**	90	100
Global disease definition	50	55.6
Diagnosis	68	75.6
Therapeutic management/drug treatment	69	76.7
Social care	52	57.8
Links/relationships with healthcare workers	39	43.3
Others *	3	3.3
**In your opinion, what are the main difficulties in accurate defining “long COVID”?**	89	98.8
The divergence/discrepancy between the different consensual definitions (WHO, FNHA)	53	59.6
Delay in onset of symptoms	39	43.8
Duration of persistence of symptoms	52	58.4
Confusion between confirmed versus probable infection in the definition	54	60.7
None	4	4.5

Abbreviations: FNHA: French National Health Authority; *n*: number; WHO: World Health Organization. * GPs reported also difficulties in ruling out a differential diagnosis such as Lyme neuroborreliosis or Chronic Fatigue Syndrome (*n* = 1), distinguishing between a confirmed long COVID, and psychosomatic symptoms and/or disorders (*n* = 1), lack of disease recognition by other specialists and healthcare workers (*n* = 1).

**Table 4:** Diagnostic difficulties of participants in patients with long COVID.

Question	All GPs (*n*)	Percentage (%)
**What could be the limiting factors in confirming or ruling out the diagnosis?**	89	98.8
Patients with a medical past history of anxiety-depressive disorders confusing symptoms	69	77.5
Several clinical features/differential diagnoses	83	93.3
Lack of training on diagnosis support (laboratory +/− imaging)	62	69.7
Difficulty in accessing tests (serology, brain [^18^F] FDG-PET/CT scan)	20	22.5
Difficulty in assessing specialist advice	34	38.2
None	0	0
Others *	4	4.4
**Which of these diseases may be differential diagnoses for “long COVID”, making your diagnosis more difficult/challenging?**	88	97.7
Irritable bowel syndrome	30	34.1
Fibromyalgia	83	94.3
Infectious disease (neuroborreliosis/mononucleosis)	44	50
Metabolic/endocrine disease (thyroid disease)	21	23.9
Organ disorders (pulmonary embolism/acute coronary syndrome/neurodegenerative diseases)	24	27.3
None	1	1.1
Others **	11	12.5

Abbreviations: [^18^F] FDG-PET/CT: Positron emission tomography/computed tomography (PET/CT) with 18 Ffluorodeoxyglucose (^18^F-FDG); *n*: number. * GPs emphasized that opinions’ disparity of specialists and reaching a conclusion is a real limiting factor which will add to patients’ anxiety (*n* = 2). Others cautioned against reinforcing a belief in a somatic entity rather than treating the underlying anxiety and/or depression that exists in some patients (*n* = 2). ** GPs have reported other diseases mimicking long COVID diagnosis: Chronic respiratory diseases (*n* = 3), psychosomatic syndrome (*n* = 3), anxiety-depressive disorders (*n* = 3), neurodegenerative diseases (*n* = 1) and exercise intolerance (*n* = 1).

**Table 5:** Therapeutic management difficulties for participants with patients with long COVID.

Question	All GPs (*n*)	Percentage of Valid Answers (%)
Do you follow-up patients with “long COVID”?	89	98.8
Yes	33	37.1
No	56	62.9
What difficulties were identified during the initial care process?	83	92.2
Lack of information documents or support on treatment	52	62.7
Difficulty in identifying patients’ complaints using diagnostic testing	67	80.7
Lack of disease recognition of “long COVID” by other specialists	41	49.4
Defining a follow-up timeline	37	44.6
Referring patients to specialist units/centers	41	49.4
No specialist unit/center in Franche-Comté	28	33.7
None	0	0
Others *	2	2.4
What difficulties did you encounter when starting treatment?	82	91.1
Several clinical features in patients with “long COVID”	53	64.6
Lack of well-defined consensus/recommendations	67	81.7
Prioritizing treatments according to clinical presentation	22	26.8
Non-financing of several treatments/drugs	9	11
Difficulty in accessing other HCWs (specialists, physiotherapists, psychologists)	37	45.1
Long waiting times for appointments with other healthcare workers	54	65.9
Management of drug interactions	1	1.2
None	1	1.2
Others	0	0
What difficulties have you experienced with patients?	83	92.2
Creating a therapeutic alliance/collaborative relationship	20	24.1
Therapeutic patient education about treatments that are not evidence-based	33	39.8
Patient non-adherence to treatment/management	13	15.7
Patient anxiety	63	75.9
Difficulties in replying to patients’ questions/queries	72	86.7
None	1	1.2
Others	0	0

Abbreviations: *n*: number; HCWs: healthcare workers. * One GP reported difficulty accessing a specialized center; another one highlighted the absence of proven treatment.

## Data Availability

Data is available on request due to privacy restrictions. The data presented in this case study are available on request from the corresponding authors.

## Supplementary Materials

The following supporting information can be downloaded at: https://www.mdpi.com/article/10.3390/jcm15082855/s1. Supplementary File S1: A multiple-choice questionnaire designed as a Google-doc-based validated questionnaire (20 questions) concerning a quantitative study of difficulties in diagnosis and therapeutic management in adult outpatients with persistent symptoms after confirmed infection with SARS-CoV-2, also known as ‘long COVID’, among general practitioners in Franche-Comté, France. Supplementary File S2: Summary sheet: Long Covid in adults; Supplementary File S3: Diagnostic criteria for Long COVID according to; Supplementary Sile S4: Geographic repartition of participants in Franche-Comté, France.

## Abbreviations

COVID-19	Coronavirus Disease 2019
GP	General practitioner
PAISs	Post-acute infection syndromes
PASC	Post-acute sequelae of SARS-CoV-2
SARS-CoV-2	Severe acute respiratory syndrome coronavirus 2
WHO	World Health Organization

## References

[1] Ochani R., Asad A., Yasmin F., Shaikh S., Khalid H., Batra S., Sohail M.R., Mahmood S.F., Ochani R., Hussham Arshad M. (2021). COVID-19 pandemic: From origins to outcomes. A comprehensive review of viral pathogenesis, clinical manifestations, diagnostic evaluation, and management. Infez. Med..

[2] Zhu N., Zhang D., Wang W., Li X., Yang B., Song J., Zhao X., Huang B., Shi W., Lu R. (2020). A Novel Coronavirus from Patients with Pneumonia in China, 2019. N. Engl. J. Med..

[3] Spiteri G., Fielding J., Diercke M., Campese C., Enouf V., Gaymard A., Bella A., Sognamiglio P., Sierra Moros M.J., Riutort A.N. (2020). First cases of coronavirus disease 2019 (COVID-19) in the WHO European Region, 24 January to 21 February 2020. Eurosurveillance.

[4] Salmon-Ceron D., Slama D., De Broucker T., Karmochkine M., Pavie J., Sorbets E., Etienne N., Batisse D., Spiridon G., Baut V.L. (2021). Clinical, virological and imaging profile in patients with prolonged forms of COVID-19: A cross-sectional study. J. Infect..

[5] Salmon Céron D., Davido B., Tubiana R., Linard F., Turgis C.T., Oustric P., Sobel A., Cheret A. (2022). Les formes prolongées de la COVID-19 ou COVID long: Formes cliniques et prise en charge. Méd. Mal. Infect. Form..

[6] Zayet S., Zahra H., Royer P.-Y., Tipirdamaz C., Mercier J., Gendrin V., Lepiller Q., Marty-Quinternet S., Osman M., Belfeki N. (2021). Post-COVID-19 Syndrome: Nine Months after SARS-CoV-2 Infection in a Cohort of 354 Patients: Data from the First Wave of COVID-19 in Nord Franche-Comté Hospital, France. Microorganisms.

[7] Soriano J.B., Murthy S., Marshall J.C., Relan P., Diaz J.V. (2022). WHO Clinical Case Definition Working Group on Post-COVID-19 Condition A clinical case definition of post-COVID-19 condition by a Delphi consensus. Lancet Infect. Dis..

[8] Horwitz L.I., Thaweethai T., Brosnahan S.B., Cicek M.S., Fitzgerald M.L., Goldman J.D., Hess R., Hodder S.L., Jacoby V.L., Jordan M.R. (2023). Researching COVID to Enhance Recovery (RECOVER) adult study protocol: Rationale, objectives, and design. PLoS ONE.

[9] Ward H., Flower B., Garcia P.J., Ong S.W.X., Altmann D.M., Delaney B., Smith N., Elliott P., Cooke G. (2021). Global surveillance, research, and collaboration needed to improve understanding and management of long COVID. Lancet.

[10] Zins M., Touvier M., Wiernik E., Lemogne C., de Lamballerie X., Blanché H., Deleuze J.-F., Villarroel P.M.S., Dorival C., Nicol J. (2022). Long-lasting Symptoms After an Acute COVID-19 Infection and Factors Associated With Their Resolution. JAMA Netw. Open.

[11] Zayet S. (2024). Brain fog across the Mediterranean. Infect. Med..

[12] Abbate G., De Iulio B., Thomas G., Priday A., Biondi-Zoccai G., Markley R., Abbate A. (2023). Postural Orthostatic Tachycardia Syndrome After COVID-19: A Systematic Review of Therapeutic Interventions. J. Cardiovasc. Pharmacol..

[13] Kamdem O.L., Guyot J., Dupre C., Gouttefarde P., Vericel M.P., Fanget M., Nkenfou C.N., Hupin D., Roche F., Botelho-Nevers E. (2025). Management of patients with post COVID-19 condition in France: A qualitative study exploring nurses’ contributions to care pathways. J. Public Health Res..

[14] Davin-Casalena B., Lutaud R., Scronias D., Guagliardo V., Verger P. (2021). French General Practitioners Frequently See Patients with Long-COVID. J. Am. Board Fam. Med. JABFM.

[15] Brennan A., Broughan J., McCombe G., Brennan J., Collins C., Fawsitt R., Gallagher J., Guérandel A., O’Kelly B., Quinlan D. (2022). Enhancing the management of long COVID in general practice: A scoping review. BJGP Open.

[16] Broughan J., Sietiņš E., Emily Siu K.Y., Clendennen N., Collins C., Fawsitt R., Lambert J.S., Savinelli S., Skeffington S., McCombe G. (2024). Enhancing long COVID care in general practice: A qualitative study. PLoS ONE.

[17] Farrell A., O’Flynn J., Jennings A. (2024). An investigation into General Practitioners’ experience with Long COVID. Ir. J. Med. Sci..

[18] Schulze J., Lind L., Rojas Albert A., Lüdtke L., Hensen J., Bergelt C., Härter M., Pohontsch N.J. (2024). German general practitioners’ experiences of managing post-COVID-19 syndrome: A qualitative interview study. Eur. J. Gen. Pract..

[19] Moreels S., Bensemmane S., De Schreye R., Cuschieri S. (2024). Caring for Long Covid patients in primary healthcare: A cross-sectional study on general practitioners’ knowledge, perception and experience in Belgium and Malta. BMC Prim. Care.

[20] Naudion P., Raffetin A., Zayet S., Klopfenstein T., Baux E., Martinot M., Piroth L., Caumes E., Chirouze C., Bouiller K. (2023). Positive intrathecal anti-Borrelia antibody synthesis: What are the implications for clinical practice? Clinical features and outcomes of 138 patients in a French multicenter cohort study. Eur. J. Clin. Microbiol. Infect. Dis..

[21] Gamillscheg P., Łaszewska A., Kirchner S., Hoffmann K., Simon J., Mayer S. (2024). Barriers and facilitators of healthcare access for long COVID-19 patients in a universal healthcare system: Qualitative evidence from Austria. Int. J. Equity Health.

[22] Wojczewski S., Mayrhofer M., Szabo N., Rabady S., Hoffmann K. (2024). “A bit of a cough, tired, not very resilient—Is that already Long-COVID?” perceptions and experiences of GPs with Long-COVID in year three of the pandemic. a qualitative interview study in Austria. BMC Public Health.

[23] Le Boedec A., Anthony N., Vigneau C., Hue B., Laine F., Laviolle B., Bonnaure-Mallet M., Bacle A., Allain J.-S. (2021). Gender inequality among medical, pharmaceutical and dental practitioners in French hospitals: Where have we been and where are we now?. PLoS ONE.

[24] Berends M.S., Homburg M., Kupers T., Meijer E.N., Bos I., Verheij R., Kuiper J., Berger M.Y., Peters L.L. (2025). Impact of Pre-Existing Comorbidities and Multimorbidities, Demography and Viral Variants on Post-Acute Sequelae of COVID-19 (‘Long COVID’) in Dutch Primary Care: A Retrospective Cohort Study. Int. J. Infect. Dis. IJID.

[25] Stahl J.P., Azouvi P., Bruneel F., De Broucker T., Duval X., Fantin B., Girard N., Herrmann J.L., Honnorat J., Lecuit M. (2017). Guidelines on the management of infectious encephalitis in adults. Méd. Mal. Infect..

[26] Conseil National de l’Ordre des Médecins (2025). Publication de L’atlas de la Démographie Médicale. https://www.conseil-national.medecin.fr/publications/actualites/publication-latlas-demographie-medicale-2025.

