# Supplementary material for: Current Difficulties for General Practitioners in the Diagnosis and Management of Long COVID Patients: A Cross-Sectional Study Assessing an Online Questionnaire
Source: J Clin Med. 2026 Apr 9;15(8):2855. doi: 10.3390/jcm15082855 (PMC13116877; doi:10.3390/jcm15082855)
Supplement: Supplementary file 1 [file jcm-15-02855-s001.zip › S1.pdf]

Supplementary material 1 : a multiple-choice questionnaire designed as a Google-doc-based validated questionnaire (20 questions) concerning a quantitative study of difficulties in diagnosis and therapeutic management in adult outpatients with persistent symptoms after confirmed infection with SARS-CoV-2, also known as 'long COVID', among general practitioners in Franche-Comté, France.
(https://docs.google.com/forms/d/1vEduI0KqD1sU5_rhJ3laCE_ktchVxrYdsGrktmoMXzQ/printform)

Consent

I agree that my data mauy be used anonymously ?
- Yes
- No

Baseline characteristics and epidemiologic data

1. Are you ?
- Female
- Male
- Do not prefer to answer

2. What is your age range ?
- Under 30
- 30 - 39 years old
- 40 - 49 years old
- 50 - 59 years old
- Over 60

3. What is your professional title ?
- Ambulatory training in primary care with supervised autonomy
- Replacement / substitute general practitioner
- General practitioner

4. Where do you practise ?
- Rural
- Semi-rural
- Urban

5. In which department do you practice ?
- (25) - Doubs
- (39) - Jura
- (70) - Haute-Saône
- (90) - Territoire de Belfort

Definitions

6. Disease recognition (of 'long COVID') ?
- Yes
- No recognition
- Lack of appropriate knowledge

7. Overall, on what issues are you experiencing / encountering considerable difficulties ?
- Disease definition
- Diagnosis
- Therapeutic management / drug treatment
- Social care
- Links / relationships with healthcare workers / professionals
- Others (please specify below)

8. If other difficulties, please specify :

9. In your opinion, what are the main difficulties in defining 'long COVID' ?
- None
- The divergence / discrepancy between the different consensual definitions (WHO, HAS)
- Delay in onset of symptoms
- Duration of persistence of symptoms
- Confusion between confirmed versus probable infection in the definition

Diagnosis

10. What could be the limiting factors in confirming or ruling out the diagnosis ?
- Patients with a medical past history of anxiety-depressive disorders confusing symptoms
- Several clinical features / differential diagnoses
- Lack of training on diagnosis support (laboratory +/- imaging)
- Difficulty in accessing tests (serology, cerebral PET scan)
- Difficulty in accessing specialist advice
- None
- Others (please specify below)

11. If other difficulties, please specify :

12. Which of these diseases may be differential diagnoses for 'long COVID', making your diagnosis more difficult / challenging ?
- Irritable bowel syndrome
- Fibromyalgia
- Infectious disease such as neuroborreliosis or mononucleosis
- Metabolic / endocrine disease such as thyroid diseases
- Organ disorders (pulmonary embolism / acute coronary syndrome / neurodegenerative diseases)
- None
- Other

13. If other suggestions, please specify :

Therapeutic management

14. Do you follow-up patients with 'long COVID'?
- Oui
- Non

15. What difficulties were identified during the initial care process ?
- Lack of information documents or supports on treatment
- Difficulty in identifying patients' complaints using diagnostic testing
- Lack of disease recognition by other specialists of 'long COVID'
- Define a follow-up timeline
- Referring patients to specialist units / centres
- No specialist unit / centre in Franche-Comté
- None
- Other

16. If other difficulties, please specify :

17. What difficulties did you encounter when starting treatment ?
- Several clinical features in patients with 'long COVID'
- Lack of well-defined consensus / recommandations
- Prioritising treatments according to clinical presentation
- Non-financing of several treatments / drugs
- Difficulty in accessing other healthcare workers (specialists, physiotherapists, psychologists)
- Long waiting times for appointments with other healthcare workers
- Management of drug interactions
- None
- Other

18. If other difficulties, please specify :

19. What difficulties have you experienced with patients ?
- Creating a therapeutic alliance / collaborative relationship
- Therapeutic patient education about treatments that are not evidence-based
- Patient non-adherence to treatment/management
- Patient anxiety
- Difficulties in replying to patients' questions / queries
- None
- Autre

20. If other difficulties, please specify :

Suggestions

21. Suggested improvements :
