# Supplementary material for: Current Difficulties for General Practitioners in the Diagnosis and Management of Long COVID Patients: A Cross-Sectional Study Assessing an Online Questionnaire
Source: J Clin Med. 2026 Apr 9;15(8):2855. doi: 10.3390/jcm15082855 (PMC13116877; doi:10.3390/jcm15082855)
Supplement: Supplementary file 1 [file jcm-15-02855-s001.zip › S2.pdf]

Summary sheet : Long Covid in adults

Epidemiology

4% French population in 2022 (2 million)
8% among patients infected with SARS-CoV-2 in 2022

- Santé publique France

Pathophysiology

Several hypotheses (multifactorial)

- Virus reservoirs
- Reactivation of underlying pathogens (EBV, HSV-1,...)
- Immune dysregulation
- Gut microbiota dysbiosis
- Vagus nerve dysfunction

Risk factors

- Femal sex
- Older age
- Underlying comorbidities : diabetes mellitus, asthma, COPD, overweight/obesity, etc...
- Severity of COVID-19 acute

Definitions

The continuation or development of new symptoms after the initial SARS-CoV-2 infection, probable or confirmed, with no other explanation

WHO "Post COVID-19 condition" = 3 months after initial infection, theses symptoms lasting for at least 2 months.

HAS "prolonged COVID-19 symptoms" = symptoms 4 weeks post-acute phase.

Symptoms

Specific : olfactory and taste dysfunction / disorders, dysautonomia (POTS)

Aspecific : chronic fatigue, postexertional malaise, brain fog, chronic cough, chest tightness, sleep discorders, concentration and/or mnesic discorders, gastro-intestinal discorders, etc...

Diagnosis

No specific diagnostic test to confirm the diagnosis

- Rule out acute-phase complications (PE, ACS, serositis, etc...)
- Decompensation of patient's comorbidities
- PASC (Postacute sequelae of SARS-CoV-2 infection) score ≥ 12
- Brain PET scan in neurological and / or cognitive presentation

Management

Personalized treatment
Multidisciplinary
Patient-centered care

- Symptomatic treatments / drugs
- Comorbidity management
- Patient information, identifying their limits (PACING approch)
- Rehabilitation
- Psychological support

Additional supports

Useful for general practionners :

HAS guidelines, PASC Score, DAC Franche Comté, infectious diseases departments, Long COVID unit.

Useful for patients :

Chabot COVID Long France on Apresj20, plateforme RAFAEL

By Cléa LE BRETON & Dr Souheil ZAYET
