# Supplementary material for: Current Difficulties for General Practitioners in the Diagnosis and Management of Long COVID Patients: A Cross-Sectional Study Assessing an Online Questionnaire
Source: J Clin Med. 2026 Apr 9;15(8):2855. doi: 10.3390/jcm15082855 (PMC13116877; doi:10.3390/jcm15082855)
Supplement: Supplementary file 1 [file jcm-15-02855-s001.zip › S3.pdf]

Supplementary material 3: Diagnostic criteria for Long COVID according to:

World Health Organization (8)	French National Health Authority (22)
-Continuation or development of new symptoms 3 months after the initial SARS-CoV-2 infection, with these symptoms lasting for at least 2 months with no other explanation.<br><br>-Symptoms may be new onset following initial recovery from acute COVID-19 episode or persist from the initial illness.<br><br>-Symptoms may also fluctuate or relapse over time.	-Confirmed or probable symptoms, with the presence of at least one of the initial symptoms beyond 4 weeks after the onset of the acute phase of the disease<br><br>-There is no explanation by another diagnosis unrelated to COVID-19.
