# Supplementary material for: Current Difficulties for General Practitioners in the Diagnosis and Management of Long COVID Patients: A Cross-Sectional Study Assessing an Online Questionnaire
Source: J Clin Med. 2026 Apr 9;15(8):2855. doi: 10.3390/jcm15082855 (PMC13116877; doi:10.3390/jcm15082855)
Supplement: Supplementary file 1 [file jcm-15-02855-s001.zip › S5.pdf]

	References	Definitions	Diagnosis	Management
Autriche	*Wojczewski S. et al.* *(32)*	-Disparity in definitions between WHO and NICE<br>-Difficulty in applying these definitions in general practice		
Irlande	*Brennan A. et al.* *(26)*<br>*Farrell A. et al.* *(28)*	-Need for a more standardized approach<br>-Specific training	-92.5% of participants did not know how to schedule the diagnosis.	
Pays Bas	*Berends MS et al.* *(34)*		-Comorbidities including mental illness = a risk factor	
Malte & Belgique	*Moreels S. et al.* *(30)*		-Lack of accurate information on diagnostic procedures	
Allemagne	*Schulz J et al.* *(29)*			-Restricted access secondary care, mental illness +++
