# Supplementary figures and images for: Current Difficulties for General Practitioners in the Diagnosis and Management of Long COVID Patients: A Cross-Sectional Study Assessing an Online Questionnaire
Source: J Clin Med. 2026 Apr 9;15(8):2855. doi: 10.3390/jcm15082855 (PMC13116877; doi:10.3390/jcm15082855)

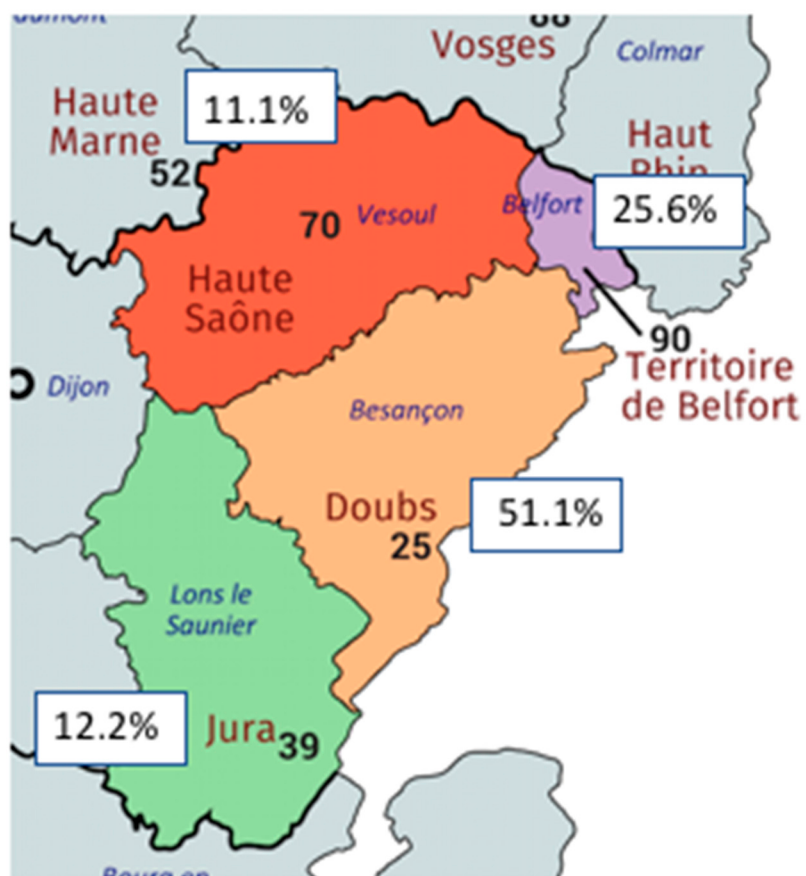

**Supplementary material 4:** Geographic repartition of participants in Franche-Comté, France

Supplement: Supplementary file 1 [file jcm-15-02855-s001.zip › S4.pdf]
